# A novel anthocyanins hydroxyethyl cellulose film for intelligent chicken meat packaging with mechanical study, DFT calculations and molecular docking study

**DOI:** 10.1038/s41598-025-10294-6

**Published:** 2025-07-26

**Authors:** Hebat-Allah S. Tohamy

**Affiliations:** Cellulose and Paper Department, National Research Centre, 33 El Bohouth Str., P.O. 12622, Dokki, Giza, Egypt

**Keywords:** Anthocyanins, Carbon nanomaterials, Sustainable films, Food biosensors, *Salmonella* detection, Color-sensing, Active packaging, Waste valorization, Environmental sciences, Chemistry, Materials science, Nanoscience and technology

## Abstract

This study presents a new type of intelligent food packaging material. The film is created using hydroxyethyl cellulose (HEC) and carbon dots that have been modified with sulfur and nitrogen (S,N–CQDs). These S,N–CQDs are produced from discarded onion peels waste, making the packaging eco-friendly and versatile for food preservation with naked eye response.. The S,N–CQDs within the HEC film (HEC-S,N–CQDs) exhibit remarkable fluorescent change when contact with *Salmonella*. When applied to chicken meat, the film effectively monitors spoilage by changing color from red to light red. This color change is attributed to the film’s pH sensitivity and its interaction with the increasing pH associated with meat deterioration. The color change which visualized by the naked-eye was an indication of the chicken meat spoilage. These make the film capable of visually detecting changes in food quality, such as spoilage, and inhibiting the growth of foodborne pathogens like *Salmonella*. Additionally, the antimicrobial properties of S,N–CQDs contribute to extending the shelf life of the packaged meat by 12 days which is more longer than the film without S,N–CQDs (3 days). DFT calculations and decreased the energy gap (0.02664 eV) prove the strong chemical reaction between HEC and S,N–CQDs within the film. The low bond length between the ligand and *Salmonella* (2.43 A°) compared to the HEC film without S,N–CQDs (2.54 A°) prove the high efficiency of antimicrobial activity for the film which containing S,N–CQDs.

## Introduction

Active food packaging plays a vital role in maintaining the integrity of the food supply chain. By safeguarding food products from spoilage, contamination, and deterioration helps to preserve product quality and extend shelf life^1–5^. Currently, food packaging relies heavily on plastics derived from petroleum. Market projections indicate a substantial increase in this sector, with estimates suggesting a growth from $362.9 billion to $534.9 billion between now and 2030, driven by an anticipated annual growth rate of 5.7%^6^. The rising reliance on synthetic food packaging materials necessitates constant technological innovation^7,8^. Unfortunately, their non-biodegradable nature harms both humans and the environment^9–11^. The urgent need to reduce plastic dependency in food processing has fueled the demand for sustainable alternatives. Sustainable packaging materials offer a promising solution to prevent the environmental impact of synthetic plastics^12^. Polysaccharides are promising biopolymers for sustainable food packaging^13–16^. Cellulose, a natural polymer, offers a strong foundation for sustainable packaging due to its abundance, biodegradability, and excellent mechanical properties^14–18^. However, films made from unmodified cellulose alone often suffer from inherent drawbacks such as poor water barrier properties, limited flexibility, and a lack of intrinsic antimicrobial activity, which restrict their application in demanding environments like food packaging^19,20^. To overcome these limitations and enhance functionality, modifications are often necessary. Therefore, we chose to utilize hydroxyethyl cellulose (HEC) for this study. HEC is a water-soluble derivative of cellulose that retains its natural advantages while offering improved film-forming capabilities, greater flexibility, and enhanced solubility, making it a more versatile and processable matrix for incorporating active components and developing advanced functional films^21^. A non-ionic water-soluble cellulose derivative, HEC has a special set of qualities that make it ideal for this use. Its exceptional film-forming properties are crucial because they enable the production of transparent, flexible, and cohesive films that can be used as efficient barriers in packaging applications. HEC is a biodegradable and eco-friendly substitute for plastics made from petroleum because it is made from renewable cellulose sources. Its natural hydrophilic properties, which is strengthened by the hydroxyethyl substitution, makes it easier to process in aqueous solutions and promotes environmentally friendly production methods. In addition to its sustainable and film-forming qualities, HEC has adjustable mechanical qualities and good compatibility with a range of additives, such as active compounds and nanoparticles. These qualities are essential for creating cutting-edge “intelligent” or “active” packaging materials that can detect spoiling or prolong shelf life^21^.

The food industry continues to grapple with the challenge of ensuring food safety and maintaining product quality throughout the supply chain. To address this, industries employ various packaging techniques, including smart, active packaging, to preserve product quality and sensing the food spoilage^2,22^. Organic dyes and metal-based quantum dots, while fluorescent, often exhibit undesirable properties like toxicity and instability. These materials can react with biological environments, leading to potential harm to living organisms. Moreover, the fluorescence of organic dyes may degrade over time, and metal-based quantum dots may require surface modifications that can compromise their fluorescence intensity and biocompatibility^23,24^. To address these limitations, we propose the use of sulfur, nitrogen carbon quantum dots (S,N–CQDs) derived from natural sources, such as onions, as a safer and more sustainable fluorescent dye alternative. Onion, a staple ingredient in the cuisine generates significant waste during both household and industrial processing. This waste includes non-edible parts such as the outer peels^25^. For this reason, instead of relying on traditional synthetic polymers for preparing S,N–CQDs, we’ll utilize Onion peel waste (OPW) as a greener solution^20^. Anthocyanins, naturally occurring colorants found in agri-food by-products, are attracting significant interest due to their valuable technological and biological properties. Their unique ability to change color in response to environmental shifts like pH known as halochromic behavior, makes them particularly useful. Therefore, using outer onion skins as a source of these bioactive and intelligent substances presents a promising opportunity for the smart food packaging industry^20,26^. Recycling anthocyanins and carbon dots from OPW by-products offers a dual benefit: it creates economic value by enabling the development of novel products while simultaneously reducing waste. This approach not only enhances the sustainability of packaging materials but also unlocks new market opportunities. Unlike conventional carbon dots, these onion-derived sulfur,nitrogen–CQDs (S,N–CQDs) exhibit naked-eye fluorescence, eliminating the need for sophisticated instrumentation^27–30^. The S,N–CQDs are promising materials for chemical sensing and pollution detection due to their unique properties: small size, luminescence, water solubility, tunability, cost-effectiveness, and ease of synthesis^20,30,31^. The sulfur nitrogen doping was found to be exhibited enhanced fluorescence emission compared to the non-doped carbon dots^32–34^.

Meat products, such as chicken, are susceptible to contamination and can serve as vectors for the transmission of pathogens^35^. The Centers for Disease Control and Prevention estimates that approximately one million people in the United States become ill annually from consuming contaminated chicken^36^. *Salmonella* contamination in chicken remains a significant public health concern^37^. Tohamy prepared a novel carboxymethyl cellulose-N-fullerene hydrogel. This material showed enhanced antibacterial activity and bacteria-induced fluorescence for biosensing. Its primary limitation is that sensing requires fluorescence equipment, not direct visual observation^38^. Javdani et al. prepared a meat sensor which *reliance on synthetic polyvinyl alcohol (PVA)* in conjunction with chitosan for the biopolymer film. While the study effectively demonstrates the enhanced physico-mechanical and functional properties, including impressive antibacterial and antioxidant activities, and excellent freshness tracking for red meat, the inclusion of a synthetic polymer like PVA somewhat *compromises the “sustainable biopolymer film” claim* to its fullest extent. Although chitosan is a natural biopolymer, the use of PVA introduces a non-biodegradable component that could *limit the overall environmental friendliness and ultimate degradability* of the film compared to a purely natural biopolymer alternative. This aspect might be a consideration for applications where 100% natural and fully biodegradable packaging is a primary objective^39^. Another study on hydroxypropyl methyl incorporated with magnetite carbon dots presents a distinct advantage over the previous carboxymethyl cellulose-N-fullerene hydrogel because it specifically addresses the need for visual detection. Unlike the earlier example where bacterial sensing was purely fluorescence-based, this research ingeniously incorporates magnetite to create a visually discernible sensor^40^. While magnetite can provide visual color and magnetic separation capabilities, a significant defect compared to using 100% natural S,N–CQDs derived from onion is the potential for environmental concerns associated with synthetic nanoparticles. In contrast, S,N–CQDs synthesized from natural sources like onions are inherently more biocompatible, biodegradable, and align better with the increasing consumer demand for truly “green” and safe food packaging solutions, minimizing any long-term health or environmental impact.

To effectively combat foodborne illnesses and reduce environmental waste, innovative antimicrobial packaging solutions that can actively detect bacterial contamination are urgently needed. In addition, the unique properties of HEC films, such as their flexibility, transparency, and biodegradability, make them a valuable material for diverse applications^37,41,42^. By incorporating S,N–CQDs into these HEC films, we can create multifunctional packaging materials with enhanced antimicrobial properties and sensitive detection capabilities. S,N–CQDs, derived from natural sources like onions, possess several key advantages include antimicrobial effects against a broad spectrum of pathogens, including *Salmonella*^21,43,44^. By embedding them within the HEC film, we can create an antimicrobial barrier that inhibits bacterial growth and extends the shelf life of packaged meat products. In addition, S,N-CQDs can bind to Gram-negative bacteria^21,22^. To illustrate the application of our approach, we will focus on *Salmonella*, a Gram-negative bacterium commonly found in contaminated chicken meat. The binding between S,N–CQDs and *Salmonella* will triggers a change in the fluorescence properties of the S,N–CQDs, enabling visual detection of *Salmonella* contamination. In addition, the change in pH (i.e. pH-sensitivity) will be detected by naked-eye color change which is an indication of food spoilage. This rapid and cost-effective method can significantly improve food safety monitoring. The United Nations’ 17 Sustainable Development Goals (SDGs) established a universal framework to tackle global issues and foster sustainable development by 2030. Food packaging significantly supports SDG 2 (Zero Hunger) by protecting food quality and reducing the pervasive problem of food waste, with an estimated one-third of all food for human consumption currently lost globally. It also aligns with SDG 12 (Responsible Consumption and Production) by offering avenues for substantial improvements in resource management. Moreover, food packaging’s hygienic properties are vital for preventing contamination, ensuring food safety, and promoting well-being, thus supporting SDG 3 (Good Health and Well-being). Furthermore, the development of intelligent or sensory food packaging contributes to climate action (SDG 13) by minimizing spoilage-related greenhouse gas emissions and reducing the overall environmental footprint of the food supply chain, while simultaneously enhancing food safety and reducing waste through real-time quality monitoring. The integration of S,N–CQDs into HEC (i.e. HEC-S,N–CQDs) provides innovative solutions that decrease environmental pollution. Utilizing HEC-S,N–CQDs in intelligent food packaging can transform supply chains, cutting energy use and greenhouse gas emissions, and thereby contributing to global climate change mitigation efforts. This approach also complements smart manufacturing and industrial automation, enhancing food product quality and safety through advanced packaging techniques^45–50^. In summary, we have successfully developed a groundbreaking intelligent food packaging film leveraging waste-derived S,N–CQDs to provide both visual spoilage indication and potent antimicrobial activity, marking a significant step towards sustainable food preservation.

## Materials and methods

### Materials

Onion peel waste (OPW), sourced from a local Egyptian kitchen, was employed to synthesize S,N–CDs. Sodium hydroxide (98%) and thiourea (99%) were purchased from Sigma-Aldrich (Saint Louis, USA).

### Preparation of sulfur, nitrogen doped carbon dots (S,N–CQDs)

To synthesize S,N–CQDs from OPW, a homogeneous solution of 4 g OPW, 9.33 g NaOH, 9.33 g thiourea, and 100 mL water was stirred, frozen, thawed, sonicated, and microwave-heated at 700 W for 7 min^30^.

### Preparation of hydroxyethyl cellulose-S,N–CQDs films

To prepare HEC-S,N–CQDs films, 1 g of HEC was dispersed in 50 mL water and agitated at 50 °C until it was entirely solved. Four different HEC solutions were mixed separately with 1:0, and 1:2 volume ratios of HEC:OPW solution with stirring for 15 min. After that, the mixtures were poured separately inside two different Teflon plates and kept at 60 °C until the film. These films are denoted as HEC-S,N–CQDs0 and HEC-S,N–CQDs2 for the 1:0 and 1:2 volume ratios of OPW solutions, respectively. The experiment was replicated twice.

### Hydroxyethyl cellulose-S,N–CQDs film application to monitor and preserve chicken meat

Fresh, boneless chicken breast meat was obtained from a local butcher shop in Cairo, Egypt. Excess blood was removed from the chicken meat by rinsing with Milli-Q water. The meat was then allowed to air dry within a laminar flow hood. Two pieces of fresh chicken meat (100 g) were wrapped with films made of HEC-S,N–CQDs0 (non-colorimetric film) and HEC-S,N–CQDs2 (colorimetric film) separately for ensuring the edges were tightly sealed by using a hot sealing machine to maximize contact between the meat and the films. Then the chicken meat was subsequently stored in at 25 °C $\pm$ 1 for duration of 12 days. Color was examined on days 0, 5, and 10.

### Characterization

#### Evaluating the pH sensitivity of intelligent films

To assess the film’s pH sensitivity, we followed the protocol outlined by Wang et al. The film was exposed to acidic and alkaline buffer solutions (pH 3 and 12, respectively) for 30 s. A smartphone was used to document any visible color changes^51^.

#### Release of anthocyanins in the HEC-S,N–CQDs2 film

To quantify anthocyanin release, the HEC-S,N–CQDs2 film was submerged in 20 mL of deionized water. This solution was then agitated at 150 rpm and maintained at 25 °C for 3 h. At predetermined time points, 3 mL aliquots of the solution were collected, and their absorbance was measured at 510 nm using an ultraviolet–visible spectrophotometer. The release rate was subsequently calculated based on these absorbance readings.

#### Mechanical properties

Film tensile properties were assessed using a LLOYD LR 10k Universal Testing Machine (England). Tensile strength (TS), elongation at break (EB%), and Young’s Modulus (Y%) for each film were determined following ASTM D-638, with a crosshead speed of 5 mm/min.

#### SEM/EDX

Microstructural characterization was performed using a Quanta/250-FEG scanning electron microscope (SEM) (Thermo Fisher Scientific, Waltham, MA, USA).

#### Fluorescence microscope

Fluorescence measurements were conducted using a Jasco FP-6500 spectrofluorometer equipped with a 150-W xenon arc lamp.

#### Fourier-transform infrared (FTIR) spectra

Fourier Transform Infrared (FTIR) spectroscopy was performed using a Mattson 5000 spectrometer (Unicam, United Kingdom). KBr pellet method was employed for sample preparation. The crystallinity index (LOI) was determined using Eqs. (1) and (2).

1 $$\text{LOI }=\frac{{A}_{1425}}{{A}_{900}}$$

2 $$\text{MHBS }=\frac{{A}_{OH}}{{A}_{CH}}$$

where A_1425_ and A_900_ refer to the FTIR absorbance at 1425 and 900 cm^-1^, respectively. In addition, A_OH_ and A_CH_ refer to the FTIR absorbance of the OH and CH peaks, respectively^40,52–56^.

#### DFT calculations

Density functional theory (DFT) calculations were carried out using the Gaussian 09 W software package. The B3LYP hybrid function was employed in conjunction with the 6-31G(d) basis set. Geometry optimization was performed using the Berny algorithm^21,57^. Different parameters were investigated via DFT calculations, including some of the optimized geometries and ground state energies, including total energy (E_T_; au), the energy of the highest occupied MO E_HOMO_ (eV), the energy of the lowest unoccupied MO E_LUMO_ (eV), the energy gap (E_g_; eV), the dipole moment (μ; Debye), the absolute hardness (η; eV), the absolute softness (σ; eV), the chemical softness (S; eV), and the additional electronic charge (ΔN_max_)^7,22^.

3 $${E}_{gap}=({E}_{LUMO}-{E}_{HOMO})$$

4 $$\upeta =\frac{({E}_{LUMO}+ {E}_{HOMO}) }{2}$$

5 $$\upsigma =\frac{1 }{\upeta }$$

6 $$\text{S}=\frac{1 }{2\upeta }$$

7 $$\Delta {N}_{max}=\frac{-\text{Pi }}{\upeta }$$

#### Antimicrobial activity

The antimicrobial activity of the films was evaluated against *Salmonella enterica* (ATCC 25566). Inoculum Preparation: Fresh overnight broth cultures of *Salmonella* were prepared in nutrient broth medium and incubated at 37 °C.

$${\text{Relative}}\;{\text{Reduction }}\left( \% \right) \, = \left( {{\text{A}} - {\text{B }}/{\text{A}}} \right) \, \times \, 100$$

where A: CFU/mL determined in untreated control sample contains pathogenic strains only without any treatment. B: CFU/mL determined in the treated sample tested.

## Results and discussion

### Mechanism of hydroxyethyl cellulose-N–CQDs film formation with the pH-sensitivity and FTIR study

**Fig. 1:**
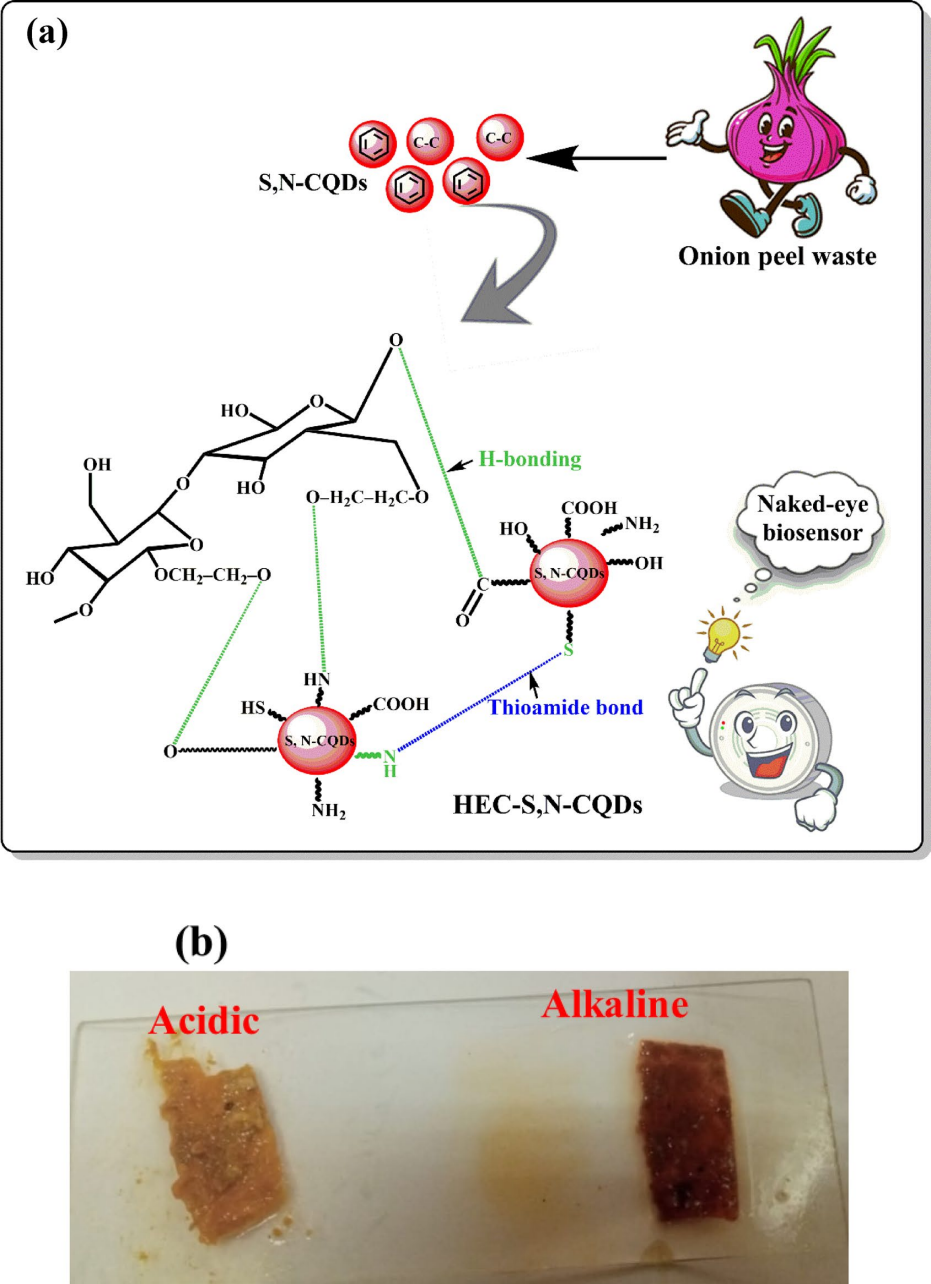

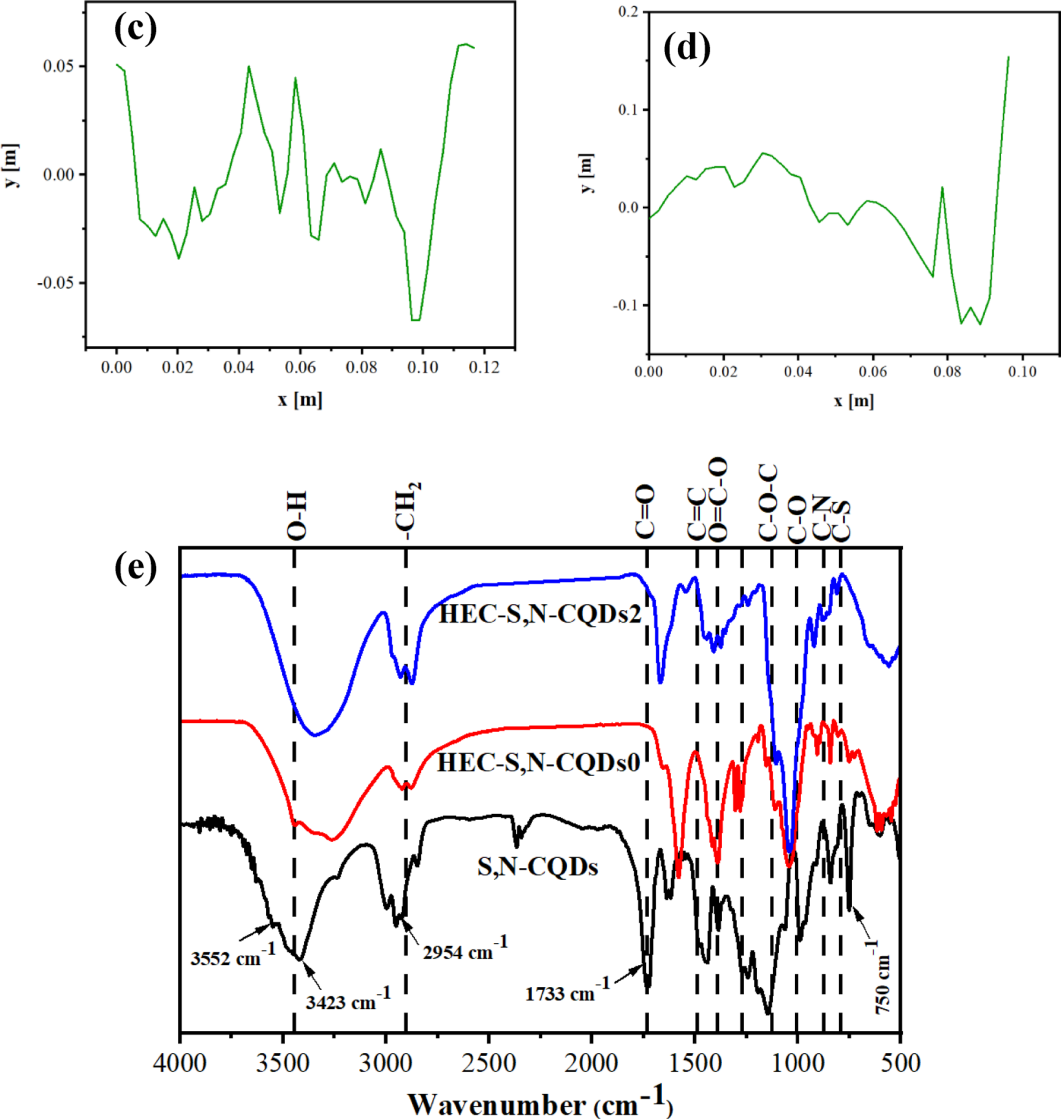
(**a**) Hydrogen bonding between HEC and S,N–CQDs for synthesizing hydroxyethyl cellulose-S,N–CQDs films, (**b**) pH-responsivity of the HEC-S,N–CQDs under acidic and alkaline medium, (**c**) roughness plot of HEC-S,N–CQDs under acidic medium, (**d**) Roughness plot of HEC-S,N–CQDs under alkaline medium, and (**e**) FTIR spectra of S,N–CQDs, HEC-S,N–CQDs0 and HEC-S,N–CQDs2.

The formation of HEC-S,N–CQDs films is driven by a complex interplay of intermolecular interactions occurring between the functional groups present on both HEC and the S,N–CQDs. These interactions are crucial for creating the cohesive and stable film structure (Fig. 1a). One primary type of interaction is hydrogen bonding. The abundant hydroxyl (–OH) groups on both the HEC polymer chains and the S,N–CQDs can readily form strong hydrogen bonds with each other. Furthermore, the hydroxyl groups of HEC can also establish hydrogen bonds with other oxygen-containing functional groups present on the S,N–CQDs, such as carboxylic acid (–COOH) groups. This extensive network of hydrogen bonds plays a significant role in integrating the S,N–CQDs within the HEC matrix, contributing substantially to the overall stability and mechanical properties of the composite films. Beyond hydrogen bonding, thiol-amine coupling reactions contribute to the cross-linking, primarily within the S,N–CQDs network itself. The sulfhydryl (–SH) groups characteristic of the S,N–CQDs can react with amine groups (–NH_2_) present on neighboring S,N–CQDs. This specific reaction involves a nucleophilic attack where an amine group donates electrons to a thiol group, resulting in the formation of a stable thioamide bond and the simultaneous elimination of a water molecule. This type of covalent cross-linking enhances the structural rigidity and robustness of the carbon dot component within the final film^58^.

We conducted a pH-sensitivity test on the HEC-S,N–CQDs2, observing a color change to faint yellow under acidic conditions and red under alkaline conditions (Fig. 1b). This means that the pH-sensitive HEC-S,N–CQDs2 film can be used to visually detect changes in pH without the need for any specialized equipment. The color change is visible to the naked eye, making it a simple and convenient method for monitoring pH levels. Beyond color change, the film’s surface morphology also demonstrated significant pH responsiveness. In acidic mediums, the films exhibited a lower roughness average (Ra) of 30.64 X 10^−3^ and a root mean square roughness (R_q_) of 27.26 X 10^−3^ (Fig. 1c). Conversely, exposure to basic mediums resulted in a higher Ra of 0.03 and R_q_ of 0.05 (Fig. 1d). This differential response in roughness could be attributed to the protonation or deprotonation of functional groups on both HEC and the S,N–CQDs. Under acidic conditions, protonation might lead to a more compact or collapsed polymer network and/or S,N–CQDs arrangement, resulting in a smoother surface. In basic conditions, deprotonation could cause polymer chain expansion or alter S,N–CQDs interactions, leading to a more open, rougher, or swelled morphology. This dual pH-responsive behavior—both colorimetric and morphological—further enhances the versatility and detection capabilities of the HEC-S,N–CQDs2 film for monitoring changes in meat quality.

Figure 1e displayed the FTIR spectra of the prepared S,N–CQDs, HEC-S,N–CQDs0, and HEC-S,N–CQDs2. The FTR spectra for S,N–CQDs showed spectra at 3552 cm^−1^ (N–H), 3423 cm^−1^ (O–H), 2954 cm^−1^ (C–H), 1733 cm^−1^ (C=O), 1617 cm^−1^ (amide I), 1440 cm^−1^ (amide II), 1384 cm^−1^ (C=C), 1247 cm^−1^ (O–C=O) and 1139 cm^−1^ (C–O–C), 989 cm^−1^ (C–O), 884 cm^−1^ (C–N), and 750 cm^−1^ (C–S)^59,60^. The N–H and C–N peaks prove the N–doping of S,N–CQDs, while, the S–H and C–S peaks prove the S–doping of S,N–CQDs. The FTIR spectra of HEC-S,N–CQDs0 and HEC-S,N–CQDs2 showed absorption peaks between 3450 and 3351 cm^−1^ (O–H), 2935–2939 cm^−1^ (Symmetric CH_2_-stretching), 2875–2877 (Anti-symmetric CH_2_-stretching), 1664–1668 cm^–1^ (C=O), 1415–1459 cm^–1^ (C=C), 1392–1409 cm^–1^ (C–O=C), 1118–1143 cm^–1^ (C–O–C), and 1044–1045 cm^–1^ (C–O)^21,28^. The HEC-S,N–CQDs2 showed additional peaks which prove the presence of S,N–CQDs at 1544 cm^–1^ (amide I), 1459 cm^–1^ (amide II), 925 cm^–1^ (C–N), and 8080 cm^–1^ (C–S). In addition the O–H group of HEC-S,N–CQDs0 was shifted from 3450 cm^–1^ to 3351 cm^–1^, which means the strong H-bonding in HEC-S,N–CQDs2. The calculated MHBS (i.e. 0.92, 0.81 and 0.87) and LOI (i.e. 1.32, 0.79 and 1.12) for S,N–CQDs, HEC-S,N–CQDs0 and HEC-S,N–CQDs2, respectively, which means high H-bonding strength between HEC-S,N–CQDs2 compared to HEC-S,N–CQDs0^34,61^. The FTIR spectrum of S,N–CQDs, HEC-S,N–CQDs0 and HEC-S,N–CQDs2 are differed as evidenced by the increased relative absorbance (RA) of the characteristic absorbance peaks of O–H (i.e. 0.91, 0.60 and 0.68) and C=O (i.e. 0.86, 0.55 and 0.79) for S,N–CQDs, HEC-S,N–CQDs0 and HEC-S,N–CQDs2, respectively. The oxygenated groups are high in HEC-S,N–CQDs2 compared to the HEC-S,N–CQDs0 due to the presence of S,N–CQDs.

### Mechanical study

**Fig. 2:**
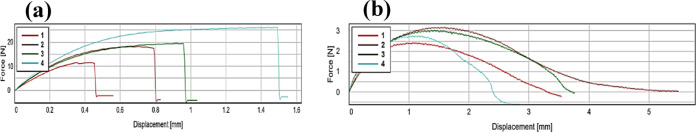
Mechanical properties of (**a**) HEC-S,N–CQDs0 and (**b**) HEC-S,N–CQDs2.

Initially, the HEC-S,N–CQDs0 had a Young’s modulus of 575.29 MPa. However, adding OPW significantly lowered this to 24.92 MPa in the HEC-S,N–CQDs2. This drop in mechanical strength is likely due to several factors. The OPW may have disrupted the film’s compact structure, weakening the cohesive forces between polymer chains. Additionally, the rigid anthocyanins in OPW could have restricted polymer chain movement, decreasing the film’s flexibility^20^. The HEC-S,N–CQDs2 film, with its higher porosity of 65.83% compared to HEC-S,N–CQDs1 at 62.01% (as detailed in the subsequent SEM section), would inherently have reduced resistance to mechanical stress (Fig. 2).

### SEM analysis: linking microstructure to film hydration and dissolution

**Fig. 3:**
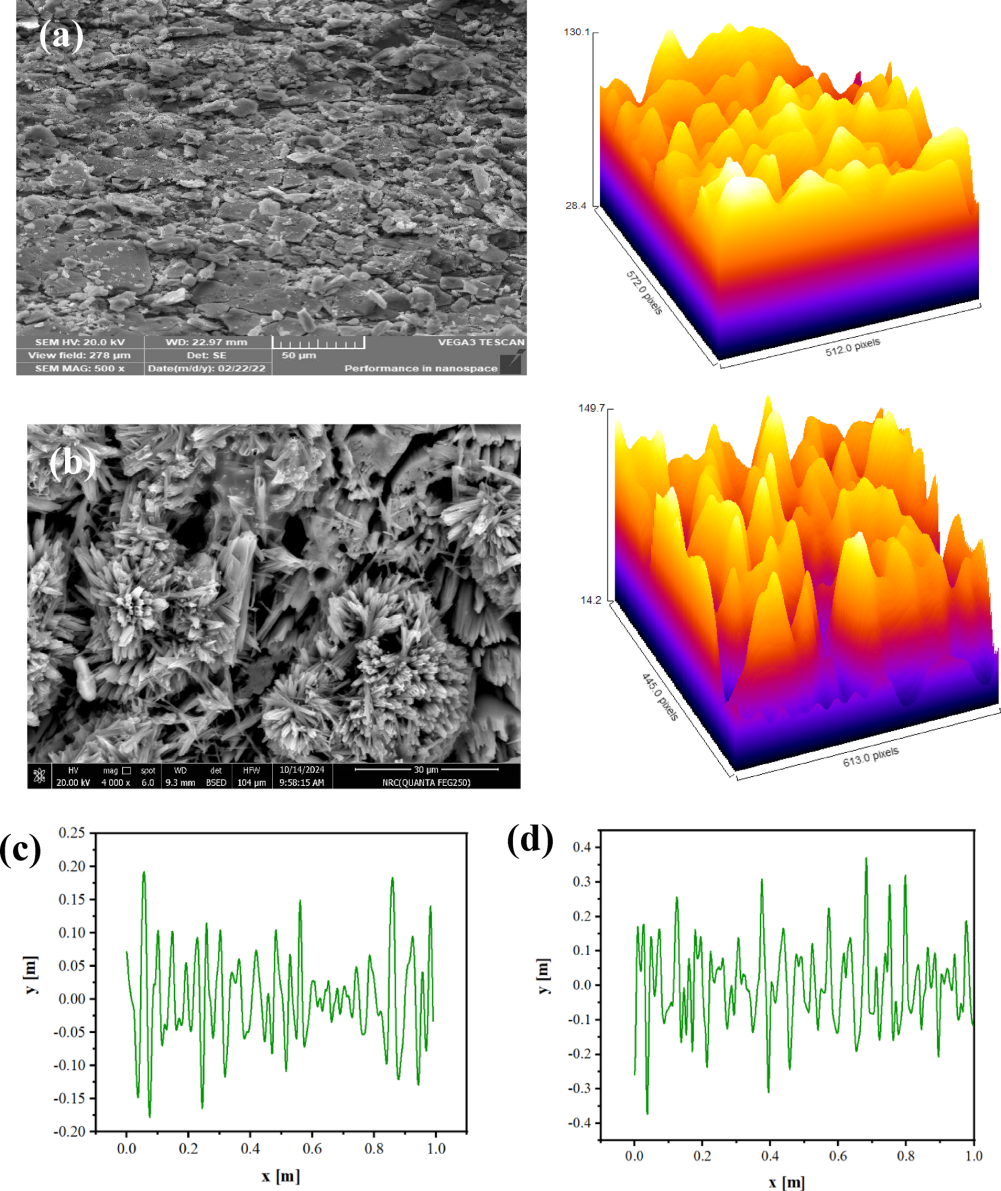
SEM analysis with 3D view for (**a**) HEC-S,N–CQDs0 and (**b**) HEC-S,N–CQDs2; roughness plots for (**c**) HEC-S,N–CQDs0 and (**d**) HEC-S,N–CQDs2.

Figure 3 illustrates the surface morphology of the HEC-S,N–CQDs0 and HEC-S,N–CQDs2 films. The HEC-S,N–CQDs0 showed a coarse and rough surface, while, HEC-S,N–CQDs2 showed a flowery shaped due to the presence of S,N–CQDs. The porosity for HEC-S,N–CQDs2 (i.e. 65.83%) is higher than HEC-S,N–CQDs0 (i.e. 62.01%). The high porosity for the sensory films is preferred because porous HEC-S,N–CQDs2 films can allow for better access of the S,N–CQDs sensor to the external environment, improving its sensitivity and response time. The incorporation of S,N–CQDs within HEC increase the surface roughness of the resulting HEC-S,N–CQDs2 composite film. The HEC-S,N–CQDs0 film showed Ra of 0.049 and R_q_ of 0.063. Upon incorporation of S,N–CQDs, these values increased to an R_a_ of 0.086 and R_q_ of 0.11 (Fig. 3c,d). This significant increase in roughness indicates a rougher surface morphology in the HEC-S,N–CQDs2 compared to the HEC-S,N–CQDs0. This enhanced roughness, which manifests as the “flowery shaped” morphology in the SEM images, indicates that the S,N–CQDs are not simply embedded but are actively influencing the film’s topography, likely forming intricate structures or altering the polymer’s arrangement during film formation. Functionally, this increased surface area is highly advantageous for sensing applications. A rougher surface offers a greater number of exposed sites for analytes from the external environment (such as volatile compounds from food spoilage) to interact with the embedded S,N–CQDs. This heightened accessibility and increased contact area are expected to significantly *improve the sensor*’*s sensitivity and accelerate its response time*, facilitating more effective detection of quality changes in products like tomatoes.

The porosity and overall structural integrity revealed by SEM are key determinants of the moisture content and water solubility of the films. For instance, the HEC-S,N–CQDs2 film, characterized by its distinct “flowery” morphology and higher measured porosity (65.83%), exhibits a more open and less dense structure compared to the HEC-S,N–CQDs0 film (62.01% porosity). This increased porosity in HEC-S,N–CQDs2 directly correlates with a greater capacity for moisture absorption. The numerous void spaces and expanded internal surface area provide more sites for water molecules to interact with and be trapped within the film matrix, explaining why this film would likely show higher moisture content. Furthermore, the same morphological differences observed in SEM are crucial for understanding the water solubility of the films. A highly porous structure, like that of HEC-S,N–CQDs2, inherently offers more pathways for water to penetrate the film’s bulk. If the film components (HEC and exposed S,N–CQDs) are water-soluble, this enhanced water ingress due to increased porosity can lead to faster dissolution or higher overall water solubility. Conversely, a more cohesive and less porous structure, even if exhibiting surface roughness as seen in HEC-S,N–CQDs0, generally presents greater resistance to water penetration and subsequent dissolution. Therefore, the microstructural details captured by SEM serve as compelling visual evidence that underpins the variations observed in the films’ moisture retention and solubility behaviors. Ultimately, these structural insights are critical for designing optimized intelligent films for sensory applications, such as the monitoring of produce like tomatoes, where controlled moisture interaction and film integrity are paramount for effective sensing performance.

### Antibacterial activity and molecular docking study

It was reported that the major spoilage chicken meat was caused by *Salmonella enterica*^62^. The findings revealed that HEC-S,N–CDs0 (denoted as 3) and HEC-S,N–CDs2 (denoted as 4) exhibited antibacterial activity by CFU method against *Salmonella enterica* equal 64% for HEC-S,N–CDs0 and 70% for HEC-S,N–CDs2. The promising anti-*Salmonella enterica* activity of HEC-S,N–CDs2 compared to HEC-S,N–CDs0. The enhanced anti-*Salmonella enterica* activity of HEC-S,N–CDs2 over HEC-S,N–CDs0 is likely attributed to the multifunctional nature of S,N-doped CDs. These materials can interact with essential bacterial components like lipids, proteins, and nucleic acids through various bonding mechanisms, including hydrogen bonding, π-π stacking, and electrostatic interactions^21,22^. Furthermore, the CDs may induce oxidative stress by generating reactive oxygen species, potentially exploiting bacterial surface proteins^63–66^.

**Fig. 4:**
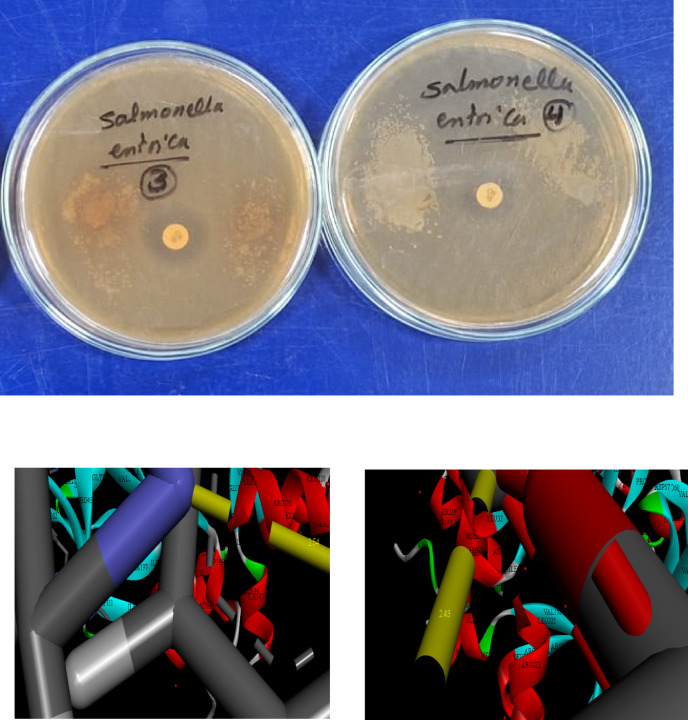
Antimicrobial activity and molecular docking study of the HEC-S,N–CDs0 and HEC-S,N–CDs2 towards *Salmonella enterica.*

Figure 4 illustrated the biological activity of HEC-S,N–CDs0 and HEC-S,N–CDs2 as a ligand against *Salmonella enterica* PDB (4YXB) as a receptor. The HEC-S,N–CDs0 and HEC-S,N–CDs2 showed binding with *Salmonella enterica* protein with bond length ~ 2.54 and 2.43 A°, respectively. This finding is consistent with experimental studies on the HEC-S,N–CDs2’s antimicrobial efficiency compared to HEC-S,N–CDs0. Shorter ligand–protein bonds enhance ligand reactivity, leading to stronger chelation, protein dysfunction, and microbial death (See Fig. 4)^21,22^.

### DFT calculations

The DFT calculations provide a robust theoretical framework for understanding both the intrinsic stability of the S,N-CQDs within the HEC matrix and their enhanced antibacterial performance. These quantum chemical parameters elucidate the electronic properties that facilitate effective interaction with bacterial cells. The μ of HEC-S,N–CQDs2 (5.196 Debye) was substantially higher than that of HEC-S,N–CQDs0 (2.640 Debye), likely due to the increased electronegativity arising from the incorporation of S, N, and O atoms from S,N–CQDs^7^. The significantly higher dipole moment of HEC-S,N–CQDs2, arising from the electronegative S, N, and O atoms, indicates increased polarity and charge separation within the composite. This enhanced polarity is crucial for antibacterial action, as it can promote stronger electrostatic interactions with the typically charged components of bacterial cell membranes, such as the lipopolysaccharides (LPS) of Salmonella. Such strong initial binding can disrupt membrane integrity, facilitating further interactions and entry of the active components.

The calculated energy gap (E_g_) for HEC-S,N–CQDs2 (0.02664 eV) was the lowest among the systems, signifying a strong chemical interaction between HEC and S,N–CQDs within the HEC-S,N–CQDs2 composite. This finding is further corroborated by the lower E_g_ of S,N–CQDs (0.2509 eV) compared to HEC-S,N–CQDs0 (0.2779 eV), suggesting the high reactivity and ease of excitation of S,N–CQDs^22^. The remarkably low E_g_ observed for HEC-S,N–CQDs2 and the S,N–CQDs themselves is central to their antimicrobial mechanism. A small E_g_ signifies that the S,N–CQDs are easily excitable, meaning electrons can readily transition from the HOMO (Highest Occupied Molecular Orbital) to the LUMO (Lowest Unoccupied Molecular Orbital). This ease of excitation, even under ambient light conditions, leads to the efficient generation of reactive oxygen species (ROS), such as superoxide radicals or hydroxyl radicals. These highly destructive ROS can cause oxidative stress, damaging bacterial lipids, proteins, and nucleic acids, ultimately leading to cell death.

The significantly higher softness of HEC-S,N–CQDs2 compared to HEC-S,N–CQDs0 indicates a greater propensity for electron transfer between the HOMO (donor) and LUMO (acceptor) states within the HEC-S,N–CQDs2 system, consistent with the lower E_g_ values^21^. This property is vital for the direct interaction of the S,N–CQDs with bacterial biomolecules. The S,N–CQDs can act as electron donors or acceptors, disrupting the redox balance within the bacterial cell or interfering with crucial enzymatic pathways through direct electron exchange. This electron transfer mechanism, alongside ROS generation, forms a powerful synergistic attack against bacterial viability, explaining the enhanced antibacterial activity observed.

**Fig. 5:**
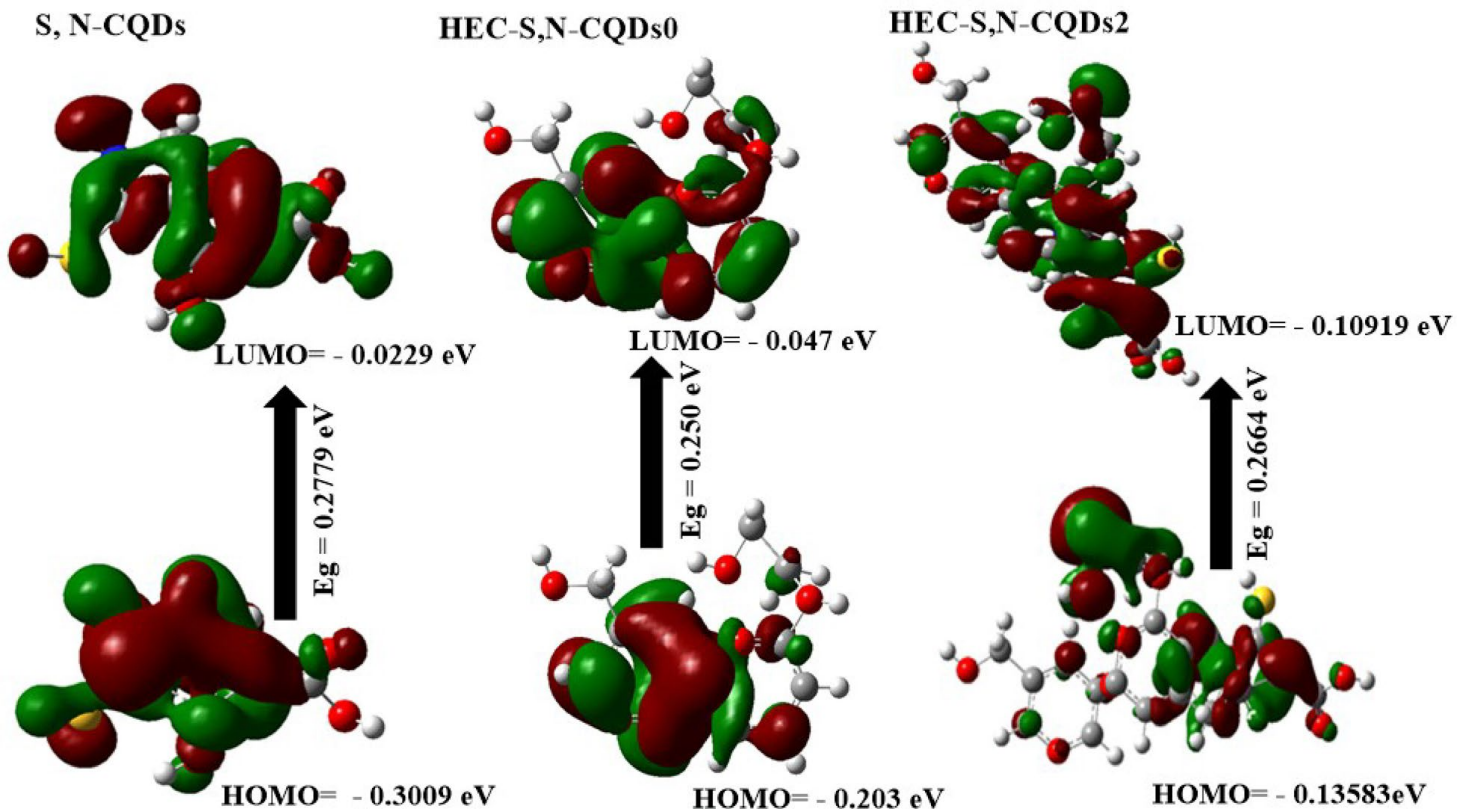
The gap energies (HOMO–LUMO) (eV) were calculated for the hydrogels using DFT B3LYP/6–31G (d), as was the molecular orbital interaction between HEC, S,N–CQDs, HEC-S,N–CQDs0 and HEC-S,N–CQDs2.

Finally, the lower E_T_ of HEC-S,N–CQDs2 (–1812.64 au) compared to HEC-S,N–CQDs0 (-939.24 au) suggests that the formation of the HEC-S,N–CQDs2 composite is energetically favorable, releasing energy during its formation. This energy release contributes to enhanced stability and resistance to bond breakage within the HEC-S,N–CQDs2 film compared to the HEC-S,N–CQDs0 film^21,22^. A stable platform is critical for sustaining the continuous release or action of the S,N-CQDs’ antimicrobial properties, thereby maintaining the film’s antibacterial performance for extended periods, which is essential for real-world food preservation (Fig. 5, Table 1).

**Table 1:** The quantum chemical parameters of S,N–CQDs, HEC-S,N–CQDs0 and HEC-S,N–. CQDs2.

DFT B3LYP/6–31G (d)	S,N–CQDs	HEC-S,N–CDs0	HEC-S,N–CDs2
E_LUMO_ (eV)	− 0.0229	0.04724	− 0.10919
E_HOMO_ (eV)	− 0.3009	− 0.2036	− 0.13583
E_g_ (eV)	0.2779	0.2509	0.02664
E_T_ (au)	− 939.24	− 941.7	− 1812.64
μ (Debye)	2.640	7.63	5.196
ɳ (eV)	− 0.1569	0.1254	− 0.12251
σ (eV)	− 6.3716	7.970	− 8.1626
S (eV)	− 0.0784	3.985	4.0813

### Fluorescence study of the hydroxyethyl cellulose-S,N–CQDs film before and after contacting with *Salmonella*

**Fig. 6:**
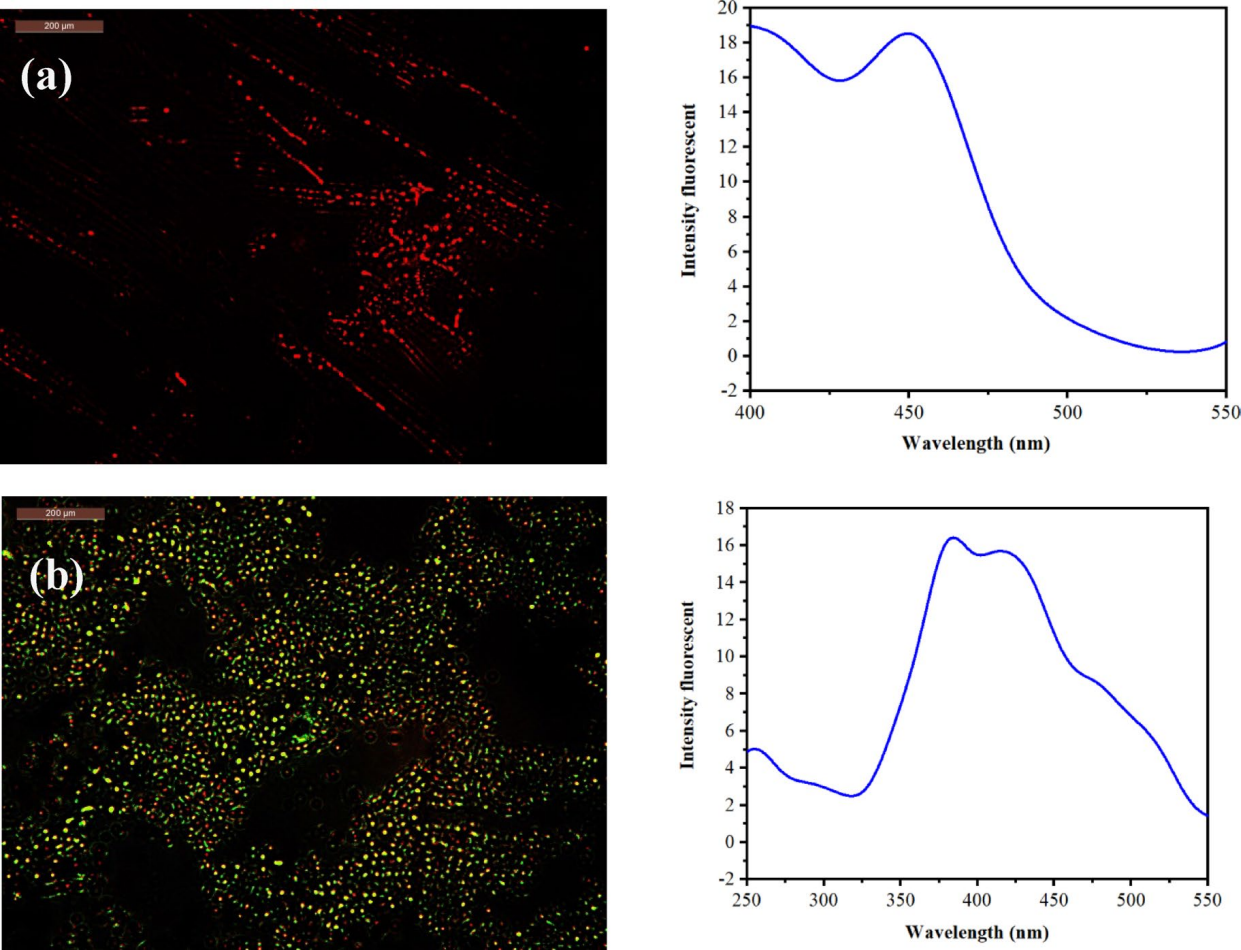
Fluorescence microscope with fluorescence intensity plots for (**a**) HEC-S,N–CQDs2 before bacterial contact, and (**b**) HEC-S,N–CQDs2 after contact with *Salmonella*.

The detailed analysis of the fluorescence intensity plots provides the quantitative basis for the visual changes observed through fluorescence microscopy. Prior to contact with *Salmonella*, the HEC-S,N–CQDs2 film exhibited a complex emission profile characterized by several distinct, smaller fluorescence peaks at emission wavelength of 450 nm. This confirms the inherent broadband photoluminescence of the S,N–CQDs within the HEC matrix, which collectively contribute to the red fluorescence visually observed under the microscope (Fig. 6a). To evaluate the effectiveness of HEC-S,N–CQDs2 in bacterial detection, we selected Salmonella as our target pathogen. *Salmonella* is a prevalent foodborne bacterium, particularly in undercooked poultry products such as chicken^67–70^. Upon interaction with *Salmonella*, a dramatic and highly specific alteration in the fluorescence spectrum was recorded, directly correlating with the qualitative shift to bright green-yellow fluorescence (Fig. 6b). This change is quantitatively underpinned by the emergence of a prominent, split peak at 384 and 420 nm, alongside the appearance of a new minor peak 254 nm. The shifting of peaks at these specific green-yellow wavelengths (384 and 420 nm) and the appearance of a new peak at 254 nm attributed to the interaction between *Salmonella*’s surface components, particularly its LPS-rich outer membrane, and the functional groups of the S,N–CQDs. This interaction likely alters the electronic states of the S,N–CQDs, leading to a modified fluorescence pathway that favors emission in the green-yellow region of the spectrum. The ability to precisely quantify these spectral changes, combined with the readily apparent visual color shift, solidifies the potential of HEC-S,N–CQDs2 films as a robust and easily interpretable fluorescent biosensor for detecting bacterial contamination^71–73^. The LPS molecules are embedded within the outer membrane and extend outward, forming a protective barrier^74^. LPS may interact with S,N–CQDs, altering their fluorescence properties and consequently modifying the emitted light color.

### Hydroxyethyl cellulose-S,N–CQDs film as achicken meat spoilage sensor by naked eye

**Fig. 7:**
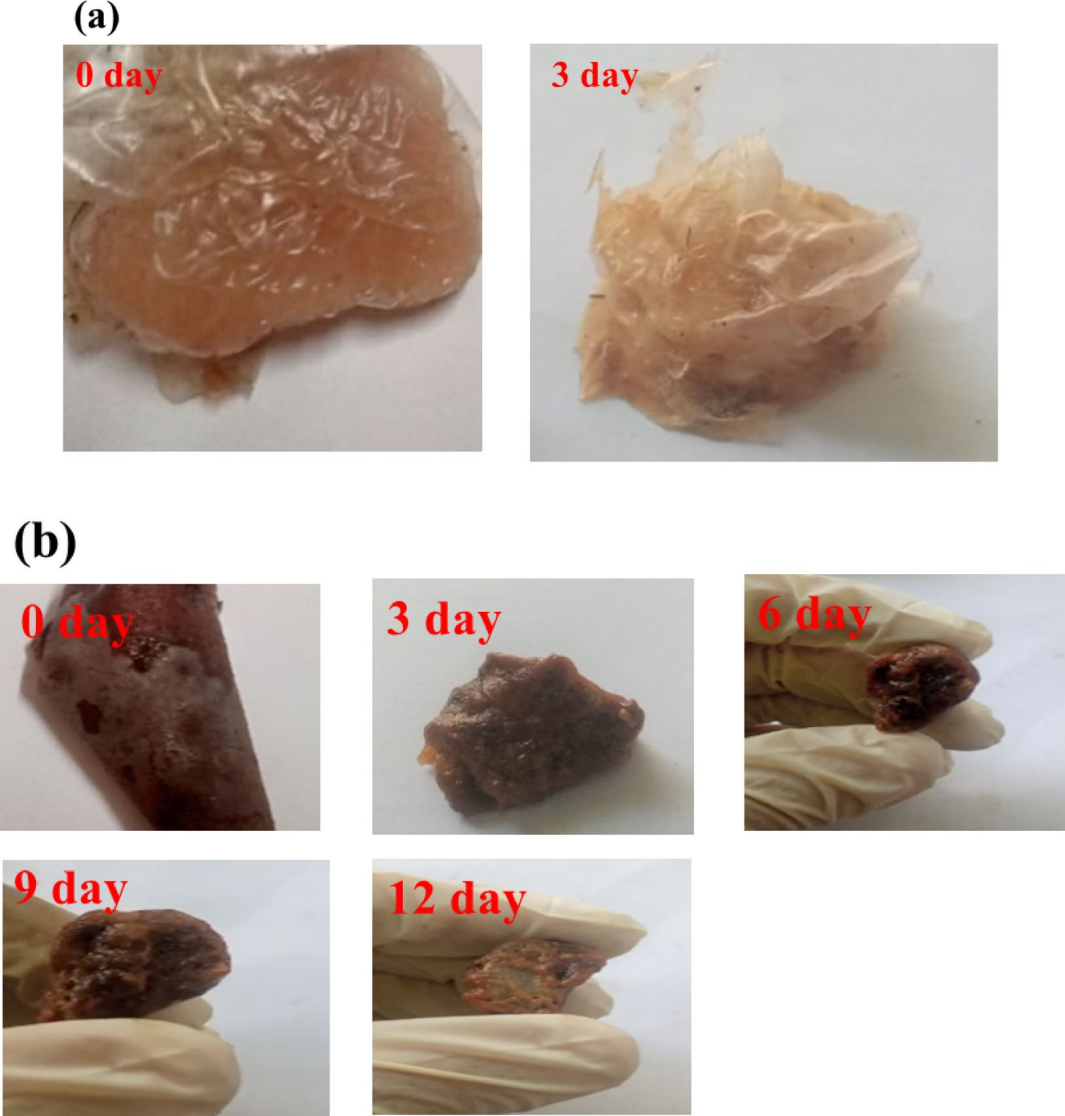
(**a**) Testing of HEC-S,N–CQDs0 on chicken meat spoilage, and (**b**) Testing of HEC-S,N–CQDs2 on chicken meat spoilage.

The pH of chicken meat typically ranges from 5.8 to 6.2. This pH level is important for the meat’s quality, texture, and flavor^75,76^. As soon as we could measure the pH of *Salmonella* inside cells (approximately 30 min post-infection), the cytoplasm was acidified from an initial pH to pH 5.65 over time^77^. The *Salmonella* is a resilient bacterium that can adapt to varying pH conditions. As it grows and metabolizes, it can produce substances that can neutralize the acidic environment, allowing it to thrive. In addition, the conversion of muscle glycogen to lactic acid during meat spoilage initially lowers the pH^78^. It is worth noting that chicken meat spoilage directly affect HEC-S,N–CQDs2’s color response (Fig. 7b), leading to substantial color change. Compared to pure HEC-S,N–CQDs0 film (Fig. 7a), developed HEC-S,N–CQDs2 film (Fig. 7b) withstand without any structural integrity loss and with a more longer period (i.e. 12 days), which might be ascribed to the antimicrobial properties of S,N–CQDs inside HEC-S,N–CQDs2. Proving the HEC-S,N–CQDs2 contention for real-time monitoring of chicken meat, the color change of the HEC-S,N–CQDs2 film from red to light red with time is linked to their sensitivity to wrapped meat spoilage (Fig. 7b).

## Conclusions

This research presents a novel approach to food packaging by developing a pH-sensitive and fluorescent film based on HEC and S,N–CQDs/anthocyanins which is derived from OPW. The S,N–CQDs, derived from OPW, offer several advantages: naked-eye fluorescence, antimicrobial properties, and pH sensitivity. The film is prepared by incorporating S,N–CQDs into an HEC matrix, creating a multifunctional material. It can be used to monitor and preserve chicken meat by visually detecting *Salmonella* contamination and pH changes, which are indicators of spoilage. The significant increase in CFU inhibition for HEC-S,N–CDs2 compared to HEC-S,N–CDs0 (70% vs. 64%), further supported by molecular docking calculations showing strong binding with Salmonella enterica protein, clearly demonstrates the film’s potent antimicrobial capabilities and its ability to extend the shelf life of packaged meat up to 12 days. These compelling analyses validate the film’s substantial potential for enhancing food safety and reducing reliance on synthetic packaging materials, offering a sustainable, intelligent, cost-effective, user-friendly, and environmentally friendly solution for monitoring food quality and preventing spoilage. These analyses validate the film’s potential for enhancing food safety and reducing reliance on synthetic packaging materials. This research offers a promising approach to sustainable and intelligent food packaging. The developed HEC-S,N–CQDs films have the potential to revolutionize food packaging by providing a cost-effective, user-friendly, and environmentally friendly solution for monitoring food quality and preventing spoilage. Future research directions could focus on enhancing the long-term stability of the film’s intelligent properties under various storage conditions (e.g., humidity, light exposure). Further comprehensive antimicrobial testing against a broader spectrum of common foodborne pathogens beyond *Salmonella*, such as *E. coli*, would strengthen its utility. Additionally, developing a more precise sensor calibration system to quantify spoilage levels or bacterial loads based on the observed colorimetric and fluorescent changes would significantly advance its practical application in the food industry. This could involve creating a standardized color chart or developing a smartphone application for automated analysis, further solidifying its position as a revolutionary tool for smart food packaging.

## Data Availability

Data is provided within the manuscript.
